# Supplementary material for: Sustained release of ancillary amounts of testosterone and alendronate from PLGA coated pericard membranes and implants to improve bone healing
Source: PLoS One. 2021 May 17;16(5):e0251864. doi: 10.1371/journal.pone.0251864 (PMC8128250; doi:10.1371/journal.pone.0251864)
Supplement: Attachment — Submitted filename: PlosOneBoneTestosteroneTherapyManuscriptReview,2-1-21.docx [file pone.0251864.s002.docx]

This study describes the controlled and sustained release of testosterone for the bone formation and its utility in bone healing. A description of the approaches for the ultrasonic application of bone promoting therapy is provided. The study concludes that the release of factors such as testosterone from these preparations promote bone repair in defect models.

There are two major concerns with the study:

1. A major issue is the inclusion of alendronate in the study with no characterization, except for one reference to its binding to bone. The half-life of a bisphosphonate on bone is long and this consideration is necessary. Osteoclast activity would be predicted to be important in the remodeling stages later in bone repair but might actually reduce bone formation early in healing. Would its diffusion in bone be comparable to testosterone in bone, as suggested at the end of the Discussion? For a conclusion of synergistic actions between testosterone and alendronate to be valid, although admittedly only implied by the authors through a reference in the Introduction, their combined effect on healing would have to be greater than the sum of their individual effects. There is no evidence presented in this study that alendronate participates in bone repair (with testosterone) as suggested in the conclusion; it is simply assumed it was released from the membrane or screw implants. As it appears that there are no alendronate (alone) controls or testosterone (alone) controls available, was there a reason that alendronate was included but not characterized in the study?
2. A better description of the data handling is necessary, especially the comparison of the testosterone treatments to the control in the microCT data. There is no mention of the normalization of the various testosterone treatments to the (100%) controls, and the reader is left to conclude it from the figure. This approach is not used for the BIC data analysis.
a) How were the controls normalized for the comparison with the testosterone treatments by microCT?
b) Additionally, with respect to this data, the increased trabecular number in the VOI-1 analysis is significant at 9 ug testosterone but there is no difference in the trabecular spacing. Likewise, the trabecular spacing in the 3 ug testosterone VOI-1 analysis is significantly reduced but there is no difference in the trabecular thickness or trabecular number parameters at this dosage. It would be expected that an increase in trabeculae would decrease their spacing for 9 ug testosterone, and a decrease in their spacing for 3 ug testosterone would be due to an increase in their number or thickness, or both. Is there an explanation for this observation, possibly related to the model or the analysis approach?

Minor issues include:

1. Insertion of the figure legends within the text of the manuscript interrupts the description it and makes it more difficult to follow.
2. The description of the anesthesia in lines 183 through 187 is unclear. Two anesthesia approaches are described. Was isoflurane inhalation used in addition to injection anesthesia?
3. Providing the dynamic bone histomorphometry in Figure 5 prior to the microCT data in Figure 4 would introduce the two-VOI approach before the figure in which it is used. The microCT analysis always precedes the histology with respect to the samples but inverting the presentation of these figures in the manuscript would clarify the two-VOI approach for the microCT analysis.

The in vitro testosterone release data is impressive and convincing, and the technology approach innovative for bone-related therapy.
